# Nondestructive Detection of *Frankia* in *Alnus glutinosa* With NIR Spectroscopy

**DOI:** 10.1002/pei3.70066

**Published:** 2025-07-04

**Authors:** Konstantinos Georgopoulos, T. Martijn Bezemer, Lars Vesterdal, Kaiyi Li, Léon de Nobel, Sofia I. F. Gomes

**Affiliations:** ^1^ Above‐Belowground Interactions Group, Institute of Biology Leiden University Leiden the Netherlands; ^2^ Department of Geosciences and Natural Resource Management University of Copenhagen Copenhagen Denmark

**Keywords:** *Alnus glutinosa*, *Frankia*, near‐infrared spectroscopy, nitrogen fixation, plant performance, spectral differentiation

## Abstract

Nitrogen (N) is essential for plant growth, yet excessive fertilizer use contributes to environmental degradation. Actinorhizal trees like *Alnus glutinosa* form symbiotic relationships with nitrogen‐fixing bacteria of the genus *Frankia,* reducing reliance on synthetic fertilizers. However, distinguishing between soil‐derived and symbiotically fixed nitrogen remains a challenge. This study investigates the potential of NIR spectroscopy as a nondestructive tool for differentiating N sources in *A. glutinosa* . Seedlings were grown in sterilized soil under controlled conditions with and without *Frankia* inoculation, and across a gradient of NH_4_NO_3_ fertilization (0–20 mM). We measured leaf chlorophyll, nitrogen content, biomass, and NIR reflectance (330–1100 nm) of the third fully expanded leaf. principal component analysis (PCA) and partial least squares (PLS) regression revealed that spectral signatures significantly differed between inoculated and uninoculated plants, particularly in the visible range around 555 nm. Despite similar leaf chlorophyll levels, *Frankia*‐inoculated plants and those fertilized with 20 mM NH_4_NO_3_ exhibited spectral differences that could otherwise not be detected by SPAD measurements. PLS regression explained up to 54.8% of spectral variance based on nitrogen source, even in the absence of unique spectral peaks. These findings highlight the potential of NIR spectroscopy for rapid, in vivo and in vitro assessment of symbiotic N‐fixation in trees, offering a novel and more precise approach than SPAD measurements.

## 1 Introduction

Nitrogen (N) is a fundamental nutrient for tree growth due to its vital role in the synthesis of amino acids, proteins, and chlorophyll, directly influencing photosynthesis and plant growth (Gong et al. 2020; Huang et al. 2021). Although plants can acquire nitrogen directly from the soil, some plants, such as black alder ( *Alnus glutinosa* (L.) Gaertn.), are able to fix atmospheric N thanks to their relationship with nitrogen‐fixing bacteria belonging to the genus *Frankia* (Orfanoudakis et al. 2004, 2009; Pujic et al. 2022). Despite these symbiotic relationships being relatively well documented (Berry et al. 1986; Carney and Matson 2005; Gentili et al. 2006; Van Der Heijden et al. 2008), it remains unclear whether N acquired from the soil or from the atmosphere via symbiotic N‐fixation can be easily and differentially detected in the foliage of the plant. Near‐infrared spectroscopy (NIR), which has been used in the past to detect plant infections by microbes via differences in wavelengths (Lim et al. 2017), could provide a promising, nondestructive method of differentiating between soil‐ and *Frankia‐*derived N in *A. glutinosa* .

The mutualistic association between *A. glutinosa* and the actinobacterium *Frankia* enables the formation of root nodules where atmospheric nitrogen is converted into a form usable by plants (Navarro et al. 2003; Pujic et al. 2019). Within the root nodules of *A. glutinosa* , *Frankia* converts atmospheric nitrogen into ammonium (NH_4_^+^), which is then supplied to the host plant (Hay et al. 2020). In return, the plant provides *Frankia* with photosynthetically derived carbon compounds, primarily dicarboxylates, to support the bacterium's energy needs (Brooks and Benson 2016; Carro et al. 2016). This nitrogen‐fixing capability allows alders and other actinorhizal plants to thrive in soils deficient in nitrogen, such as degraded lands (Diagne et al. 2013), thereby facilitating ecological succession in such environments (Benson and Silvester 1993). However, past studies have shown that high levels of available N in the soil can lead to similar plant performance as when the plant establishes a symbiosis with *Frankia* (Ballhorn et al. 2017). Therefore, it becomes challenging to disentangle the N source (through symbiont or soil) and therefore quantify the relative contribution of N sources to tree growth.

Plant spectral data encapsulate a wide range of information about both the structure and physiological processes of plants (Kothari and Schweiger 2022). By analyzing reflectance spectra from leaves, scientists can deduce various plant characteristics (Ustin and Jacquemoud 2020) such as leaf chlorophyll content (Danh et al. 2021) and detect plant stress (Asner et al. 2016), natural enemies (Sapes et al. 2022) and microbial infections (Lim et al. 2017). N taken up directly from the soil and N derived from the atmosphere via *Frankia* N‐fixation are two different pathways for N to enter the plant, and as such, they could reflect differently in the visible or infrared light spectra. Although conventional inspection of *Frankia* symbiosis is carried out by directly scavenging the roots of actinorhizal plants for nodules, NIR methods could provide a nondestructive alternative that has not been explored before.

In this study, we assess whether NIR spectroscopy can be used to differentiate between N provided through fertilization and N fixed symbiotically by *Frankia*. To investigate whether soil‐ or *Frankia‐*derived N is reflected differently in the NIR spectra, trees were grown in sterilized soil, across gradients of increasing available N (NH_4_NO_3_) and in the absence or presence of *Frankia*. NIR spectra were obtained from the leaves of trees. We hypothesize that *Frankia‐*inoculated plants perform similarly, in regard to plant biomass, to uninoculated plants that receive enough N fertilizer, and that the N derived from *Frankia* will be reflected via unique spectral peaks in the NIR wavelengths compared to soil‐derived N.

## 2 Materials and Methods

This study aimed to distinguish between N supplied through fertilization and N symbiotically fixed by *Frankia* using NIR spectroscopy. Black alder seeds were sourced from the Dutch State Forestry Department, Staatsbosbeheer. To prepare the inoculum for all the experiments, local field nodules were collected from the roots of a single population of *A. glutinosa* trees growing naturally in a semiforested area in Leiden, Netherlands (Latitude: 52.171515 N, Longitude: 4.468883 E). Fresh nodules were collected on the same day as each experiment was set up, ensuring that only healthy, intact nodules of mature *A. glutinosa* trees were used for inoculum preparation.

### 2.1 Seed Germination

To minimize external contamination and ensure that *A. glutinosa* seedlings were only inoculated with *Frankia*, we germinated seedlings under sterile conditions. To achieve this, *A. glutinosa* seeds were surface‐sterilized by shaking for 20 min in a 14% bleach solution and placed on 0.5 Murashige and Skoog (MS) medium (Kahrizi et al. 2018) agar plates for germination. The plates were sealed with parafilm and incubated vertically in a controlled growth cabinet with a 16:8 light‐to‐dark cycle at 20°C during the light phase and 17°C during the dark phase. Seedlings were allowed to germinate and develop for 4 weeks until they had at least two mature leaves before being used in experiments. To maintain seed surface sterility, plates were inspected daily for signs of bacterial or fungal contamination near the seeds. If contamination was detected, uncontaminated seedlings were carefully transferred to new sterile agar plates under a flow hood, while contaminated seedlings and plates were discarded. Additionally, this method allowed for the selection of seedlings with consistent shoot and root sizes, providing greater uniformity for experimental setups.

### 2.2 Fertilization vs. *Frankia* Derived N Experiment

An experiment was established to investigate the hypothesis that N derived from *Frankia* will be reflected via unique spectral peaks in the NIR spectra compared to soil‐derived N. Four weeks after germination, as previously described, seedlings were transferred into 1 L pots (dimensions: 11 × 11 × 12 cm). To ensure that any differences in the spectra were solely due to the N source and not other stress factors, all the pots were filled with the same gamma‐sterilized grassland soil for which soil properties were analyzed (see Georgopoulos et al. 2025 for details). This soil was predominantly sandy, comprising 98% sand with only 1.4% fine particles (silt and clay) and a minimal gravel content of 0.6%. Nutrient availability was limited, with moderate to low concentrations of nitrogen (NH_4_^+^ at 37.82 ± 1.61 mg/kg and NO_3_^−^ at 15.41 ± 0.57 mg/kg). Phosphorus levels were also low, as indicated by a PO_4_^3−^ concentration of 3.86 ± 0.41 mg/mg. The soil exhibited a slightly alkaline reaction, with a pH of 7.37 ± 0.02, and contained a low amount of soil organic matter (1.85% ± 0.04%). To create a gradient of N availability, pots were fertilized with solutions containing 0, 1.25, 2.5, 5, 7.5, 10, or 20 mM NH_4_NO_3_ (Sigma Aldrich). While *Frankia* provides NH_4_^+^ to the plant via N‐fixation (Hay et al. 2020), in most soils, plants encounter a mixture of NH_4_^+^ and NO_3_^−^ and can uptake both. As such, both forms of N were supplied, ensuring that non‐nodulated (pure) controls and nodulated (*Frankia*‐inoculated) plants all have access to the two major soil‐available N sources and preventing pH‐driven nutrient imbalances (rhizosphere acidification with NH_4_^+^, alkalinization with NO_3_^−^) that themselves may alter plant metabolism and hence, potentially, NIR spectra. Each pot received 15 mL of the designated solution twice weekly for a period of 12 weeks. For each nutrient level, 20 pots were prepared, half of which were inoculated with *Frankia* while the remaining half served as uninoculated controls (*n* = 10 replicates). The *Frankia* inoculum, prepared fresh on the day of the experiment, was made by crushing surface‐sterilized *Frankia* nodules and homogenizing nodule tissue in autoclaved Milli‐Q H_2_O (100 mg / 1 mL; Ballhorn et al. 2017). After fertilization, 1 mL of the homogenate was pipetted into a small indentation near the roots of the inoculated seedlings. To ensure that the mechanical insertion of the inoculum did not influence plant performance, 1 mL of MilliQ H_2_O was similarly pipetted near the roots of noninoculated seedlings. Due to space limitations, the experiment was performed in two sets, one set including the 1.25–5 mM fertilizers and the other set including the 7.5–20 mM fertilizers. The two sets were performed using the exact space in the same growth chamber and the exact light and humidity conditions, but 12 weeks apart, and included their own sets of controls. As such, per experimental set, 10 pots with sterilized soil were left untreated (neither inoculated nor fertilized, which we call control pure), while another 10 pots received only the *Frankia* inoculum (henceforth known as nodulated control), bringing the total number of pots to 160. To ensure that differences in seasonal variations and light conditions would not influence the outcome of either experimental set, plants were grown in a climate‐controlled room with a relative humidity of 70%, a 16‐h light and 8‐h dark cycle, and temperatures of 20°C during the light phase (LED lights; Valoya LightDNA BX120, NS1 + FR, 2‐channel) and 17°C during the dark phase. Light intensity (%) changed according to a timed schedule (7:00, 0%, 8:00, 30%, 11:00, 80%, 13:00–17:00, 100%, 19:00, 80%, 22:00, 30%, 23:00, 0%). Pots were watered three times per week to soil saturation. Over the course of 12 weeks, plant growth metrics, including stem height and leaf count, were recorded weekly. Leaf chlorophyll was recorded from the fourth until the final week of the experiments using a Chlorophyll Meter SPAD‐502Plus (Konica Minolta Sensing Europe B.V.). Prior to unpotting, on the day of the harvest, NIR spectra were measured from the third leaf from the top of each plant (the same leaf used for each chlorophyll measurement) using an Inventech Benelux NIR spectrometer (Oosterhout, Netherlands) with a spectral detection limit of ~330–1100 nm. Spectra were taken from fresh, intact leaves on live plants, and no grinding or drying was performed prior to spectral measurements. We selected the third leaf from the apex because it consistently represented the youngest fully expanded leaf across all treatments. Previous studies have shown that once *A. glutinosa* leaves reach full expansion, their pigment and nitrogen concentrations remain relatively stable for an extended period (Starčević et al. 2024). By sampling a fixed leaf position, we standardized for developmental stage rather than chronological age, thereby minimizing the potential influence of treatment‐induced differences in growth rate on the NIR spectra. Prior to measuring the first leaf and for every 40 measurements after that, a blank measurement was taken. NIR data were preprocessed by correcting based on the blank measurements by removing spectra lower than the blank. In total, one NIR measurement was taken from each fresh leaf of each plant for a total of 160 collected samples (80 from the first and 80 from the second set).

At harvest, plants were carefully unpotted, and roots were rinsed under running tap water. Root nodules were counted, excised using a razor blade, and oven‐dried at 40°C to determine the total root nodule biomass. The remaining plant biomass was divided into stems, leaves, and roots. All components were oven‐dried under the same conditions to determine aboveground, belowground, and nodule biomass. For leaf nitrogen content analysis, oven‐dried leaf samples were ground using a QIAGEN TissueLyser II Bead Mill (Hilden, Germany) at 370 rpm for 5 min. Leaf nitrogen percentages were quantified using the dry combustion method (Matejovic 1997) with a Thermo Scientific FLASH 2000 CN analyzer (Milan, Italy).

Soil NH_4_^+^‐N and NO_3_^−^‐N concentrations were determined using a standard 1 M KCl extraction and spectrophotometric analysis (Kachurina et al. 2008), while PO_4_^3−^‐P concentrations were measured using an extraction method with 0.01 M CaCl_2_ (Houba et al. 2008). Nutrient concentrations were expressed as mg of NH_4_^+^‐N, NO_3−_‐N, and PO_4_^3−^‐P per kg of soil (Table S1).

### 2.3 Statistical Analysis

#### 2.3.1 Fertilization vs. *Frankia*‐Derived N Experiment

The height of the plants in the control treatments differed between the sets at the time of the harvest (Set 1: Nodulated: 33.09 +/− 1.99, Pure: 24.70 +/− 1.23; Set 2: Nodulated: 38.76 +/− 2.15, Pure: 19.65 +/− 1.00). As such, to make the two experimental sets comparable, and to assess chlorophyll levels relative to the pure (uninoculated, unfertilized) control, we adjusted the chlorophyll measurements by subtracting the average chlorophyll value of the pure control for each experimental set and week from the corresponding measurements. For each week, a linear model (LM) was used to evaluate the effects of *Frankia* inoculation (No *Frankia*, *Frankia*) and fertilizer concentration (0, 1.25, 2.5, 5, 7.5, 10, and 20 mM) on leaf chlorophyll (see Tables S2 and S3), using the *lme4* R package (v1.1–35.1; Bates et al. 2015). Model residuals were assessed for normality through the Shapiro–Wilk test, a QQ plot, and a histogram to visually inspect skewness.

To examine whether *Frankia* inoculation (No *Frankia*, *Frankia*) and fertilization concentration (0, 1.25, 2.5, 5, 7.5, 10, and 20 mM) impacted nodulation by the plants, a LM was used. Nodule biomass was square‐root transformed to improve the normality of the data. To assess how closely each fertilizer treatment replicates the performance of *Frankia* inoculation, we quantified how closely each uninoculated fertilizer treatment matched the nodulated control by subtracting the control's mean harvest values of each experimental set for aboveground biomass, leaf chlorophyll, and leaf nitrogen (%) from those of the corresponding uninoculated treatments. One‐sample t‐tests were then performed to compare the means of each uninoculated, fertilized treatment to zero (nodulated control performance) using the *dplyr* R package (v2.5.0). To account for multiple comparisons, *p* values were adjusted using the false discovery rate (FDR) with the Benjamini–Hochberg method (Table S4).

#### 2.3.2 NIR Spectra

Preprocessing of spectra was done using standard normal variate (SNV) transformation to correct for scatter effects and to normalize across samples, as implemented in the *mdatools* package (v.0.14.2; Kucheryavskiy 2020). To examine dissimilarities between the spectra of each treatment, a principal component analysis (PCA) was conducted using the *factoextra* package (v.1.0.7; Kassambara and Mundt 2017) based on Euclidean distances in order to account for the negative values after normalizing. Differences between treatments were evaluated through Permanova using the *vegan* package v2.6–4 (Oksanen 2015). Pairwise comparison tests were conducted with the *ecole* package v.0.9–2021 when significant differences were observed in the Permanova. Finally, partial least squares (PLS) regression models were used to assess whether NIR spectra could predict the nitrogen source (*Frankia* vs. fertilization). To evaluate model performance and avoid overfitting, we used leave‐one‐out cross‐validation (LOOCV), where each observation was excluded once as a validation sample while the model was trained on the remaining data. This approach was implemented using the *mdatools* package in R. The number of latent variables was selected based on the minimum root mean square error of prediction (RMSEP) across iterations. Model performance was evaluated using the proportion of variance explained (*R* ^2^) and RMSEP.

## 3 Results

### 3.1 Plant Performance

**FIGURE 1:**
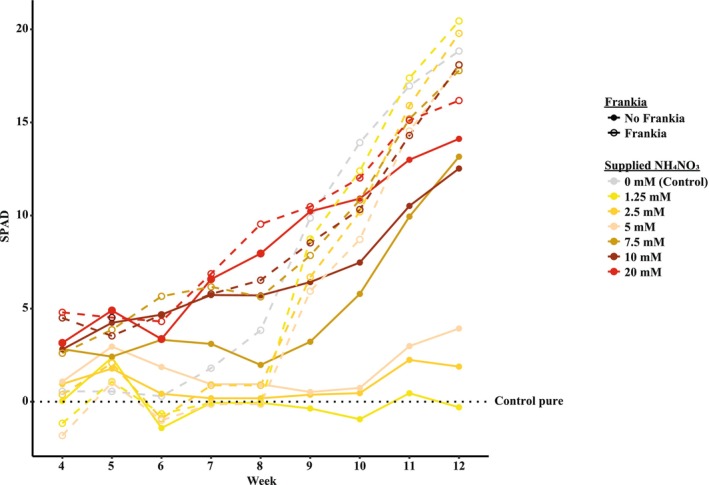
The effects of *Frankia* inoculation (*Frankia*, No *Frankia*) and fertilizer level (0, 1.25, 2.5, 5, 7.5, 10, and 20 mM) on leaf chlorophyll levels per week. Measurements begin at week 4 as the leaves were too small to measure nondestructively during the first 3 weeks. To make the two experiments comparable, the average chlorophyll value of the uninoculated control (control pure) for each experiment and each week was subtracted from the corresponding chlorophyll measurements of that week. As such, everything above or below the zero‐line reflects a better or worse performance than the control pure, respectively. The values were calculated from *n* = 10 replicates. Error bars have been omitted to enhance visual clarity but the means and standard errors can be found in Table S5.

Both *Frankia* inoculation and fertilizer concentration had a significant interaction effect on nodule biomass (DF = 6, F = 11.726, *p* < 0.001). Plants inoculated with crushed nodules and fertilized with 20 mM NH_4_NO_3_ developed almost no nodules, suggesting that high nitrogen availability suppresses nodulation. In contrast, inoculated plants receiving any of the lower fertilizer concentrations produced nodule biomass comparable to the nodulated control. As expected, all plants that did not receive the *Frankia* inoculum did not form nodules (Figure S1). *Frankia* inoculation had a significant effect on leaf chlorophyll levels from the 9th until the 12th week of plant growth (Figure 1; Table S2). From the 4th until the 8th week of plant growth, chlorophyll levels were indistinguishable between inoculated and uninoculated plants (Figure 1; Table S2). When excluding the *Frankia* inoculated treatments, fertilizer concentration had a significant effect on leaf chlorophyll levels at every week of measurement (Table S3). However, only plants that were fertilized with 10 and 20 mM NH_4_NO_3_ exhibited significantly higher leaf chlorophyll levels than the control pure (uninoculated and unfertilized) after week 6 and 7.5 mM after week 9 (Figure 1).

At the time of the harvest, trees fertilized with 10 mM NH_4_NO_3_ exhibited similar aboveground biomass to the nodulated control but had significantly lower leaf chlorophyll and nitrogen content (Table S4; Figure S2). In contrast, trees receiving 20 mM NH_4_NO_3_ produced 47% more biomass, maintained comparable leaf chlorophyll levels, and exhibited only a 19% reduction in leaf N relative to the nodulated control. Fertilizer treatments below 10 mM NH_4_NO_3_ resulted in significantly lower biomass, chlorophyll, and leaf N content than the nodulated control (Table S4; Figure S2). Since subsequent NIR measurements were conducted on leaves, the 20 mM NH_4_NO_3_ treatment was considered the closest to *Frankia* inoculation in terms of plant performance as SPAD showed no difference. Thus, it was used for direct comparison between the NIR spectra to disentangle the effect of fertilizer addition and *Frankia*‐fixed N.

### 3.2 Spectral Characteristics of *Frankia*

**FIGURE 2:**
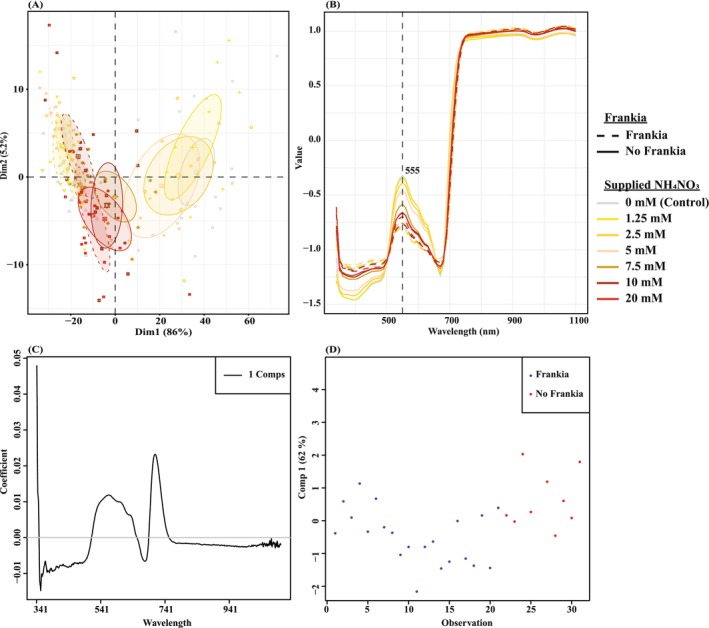
(A) Principal components analysis showing the dissimilarity between the (near infrared) NIR spectra of the whole dataset after standard normal variate (SNV) correction. Ellipses represent Euclidean distances at the 95% confidence level. (B) The aggregated NIR spectra of the whole dataset after SNV correction ranging from 341 to 1100 nm with visible maximum absorbance peaks linked to the purpose of the study at 555 nm. The y‐axis is the normalized reflectance after SNV correction. (C) The coefficient plot and (D) score plot of the PLS model including the NIR spectra of only the nodulated control against the similarly performing 20 mM fertilized and uninoculated treatment using 1 component.

In total, 121,440 raw reflectance spectra were measured from 341 to 1100 nm from the leaves of 160 plants. Rising peaks were observed at 500 nm, reaching the highest average reflectance, where differences between treatments are visible for that peak range at 555 nm and decreasing henceforth until 673 nm. Reflectance of *A. glutinosa* leaves for this range starts to plateau at 750 nm and reaches its maximum at 1100 nm (Figure 2B). At 555 nm, *Frankia* inoculation had a significant effect on the reflectance (ANOVA: Df = 1, F = 4.40, *p* = 0.04) and the reflectance of the 20 mM uninoculated treatment was significantly higher than the nodulated control, suggesting a lower N concentration.

There were significant differences between the spectra of the different individual treatments (Permanova: pseudoF = 26.16, *R* ^2^ = 0.69, *p* = 0.001) as observed in the PCA plot (Figure 2A). Specifically, pairwise comparisons revealed the NIR spectra of the *Frankia*‐inoculated treatments were significantly different from all the uninoculated treatments except for those fertilized with 10 and 20 mM (Figure 2A). A significant dissimilarity was also observed when grouping the plants based on *Frankia* inoculation (Figure S3; Permanova: pseudoF = 133.54, *R* ^2^ = 0.46, *p* = 0.001). When explicitly comparing the nodulated control with the similarly performing 20 mM fertilized and uninoculated treatment, these were more similar but still significantly different (Figure S4; Permanova: pseudoF = 6.68, *R* ^2^ = 0.18, *p* = 0.003), showing that despite the similar plant performance, differences between treatments could still be detected in the NIR spectra.

PLS analysis on the whole dataset (1 component based on lowest RMSEP; Figure S5A) showed that the presence of *Frankia* could explain 54.8% of the observed variance, while PLS analysis on the NIR spectra of only the nodulated control against the similarly performing 20 mM fertilized and uninoculated treatment (1 component based on lowest RMSEP; Figure S5B) showed that the presence of *Frankia* could explain 31.2% of the observed variance. No visual differences were observed in the spectral peaks between the two datasets (Figure 2C; Figure S6A).

## 4 Discussion

In this study, we investigated the potential use of NIR spectroscopy for nondestructive detection of N fixed by *Frankia* in *A. glutinosa* . The spectral differences observed between the two N sources indicate that biological N fixation has a measurable impact on leaf reflectance, even when plants exhibit similar chlorophyll levels when supplied with high levels of soil N.

A key finding from our study is that despite the similarities between plants inoculated with *Frankia* and plants fertilized with 20 mM NH_4_NO_3_—reflected mainly by their chlorophyll content, their spectral signatures were distinguishable. This suggests that symbiotic N‐fixation influences leaf biochemistry and structure in a way that is detectable using NIR spectroscopy. Specifically, the reflectance values at 555 nm differed significantly between the nodulated control and the plants fertilized with 20 mM NH4NO3. It is important to mention that the 555 nm peak falls within the common visible spectral region of chlorophyll absorption for green leaves (400–720 nm; Gitelson et al. 2022; Raddi et al. 2022) and thus it is surprising that two treatments with indistinguishable chlorophyll levels had significantly different reflectance. As such, these differences may be attributed to the higher leaf N of the *Frankia* inoculated treatments, or subtle structural changes in leaf tissue and pigment following symbiotic N‐fixation, although the latter is unlikely considering the lack of detectable differences in physical leaf properties in this and other studies that used *Frankia* (Arnebrant et al. 1993; Ballhorn et al. 2017; Vincent et al. 2025). It could also simply mean that the NIR meter can more precisely detect slight differences in N that are not detected by the SPAD meter which measures the spectral absorbance of chlorophyll only in the red (600–700 nm) and near‐infrared region, accounting for only part of the 500–670 nm range where we observe rising peaks for our treatments. This is in partial agreement with our initial hypothesis as we were able to detect significant differences between the spectral signatures and reflectance of inoculated and noninoculated but similarly performing plants, even though these were not owed to any unique spectral peaks in the measured region (341–1100 nm). Past studies have shown that infection by microbes (e.g., *Fusarium graminearum* and *Fusarium asiaticum*) was able to be detected using NIR methods based on differences in the reflectivity intensity despite a lack of unique spectral peaks for infected plants (Lim et al. 2017). To our knowledge, this is the first study that has attempted to differentiate between *A. glutinosa* plants inoculated with *Frankia* and uninoculated plants using NIR techniques.

The addition of crushed nodules generally improved plant performance across most measured parameters, particularly at lower N concentrations. Previous studies have shown that high soil N can inhibit nodulation (Arnone III et al. 1994), and we observed a similar trend: nodulation was nearly absent at the highest fertilizer level (20 mM), even with crushed nodule inoculation, whereas it still occurred at lower N levels (1.5–5 mM). These results suggest that at low N levels, nodulation is not suppressed, allowing N fixation to compensate for limited soil N. In contrast, at high fertilization levels, nodulation is inhibited, but plants still perform well due to the abundance of readily available N in the soil. Additionally, these results identify 20 mM as the threshold of nitrogen addition required to inhibit nodulation.

While our study demonstrates the feasibility of using NIR spectroscopy to detect *Frankia*‐derived N by more accurately detecting differences in leaf N, several limitations must be considered. First, the spectral differences between *Frankia*‐inoculated and high‐N fertilized plants, although significant, did not reveal different peaks characteristic of the presence of *Frankia* within this spectral region but only a difference in reflectivity. Previous studies have demonstrated that *Frankia*‐induced nodulation influences the levels of citrulline, ornithine, and glutamine in nodules and roots, where their concentrations are notably higher compared to those in the leaves (Arnebrant et al. 1993; Miettinen and Virtanen 1952). It is possible that adding NH_4_^+^ to the soil masks the distinct signature of symbiotic N‐fixation since the plant assimilates ammonium from both soil and fixation through similar biological pathways (Ohyama and Kumazawa 1980). Additionally, the high percentage of variation explained by latent variables suggests that additional physiological and biochemical factors, beyond nitrogen source alone, contribute to spectral variation. While other leaf constituents, such as secondary metabolites, could potentially be affected by nodulation (Brooks and Benson 2016), no other regions in the spectrum were found to vary significantly. Lastly, although we were able to detect a significant spectral difference between the 20 mM and nodulated control at 555 nm, field testing should be done to confirm robust discrimination in practical applications. Our proof of concept was carried out under highly controlled conditions with a single tree species and a uniform soil background and relied on multivariate differences in overall reflectance rather than on any distinct peaks. In more heterogeneous natural environments—where temperature, canopy cover, inter and intraspecific variation, soil background, and light exposure all introduce additional spectral variance—the subtle reflectance offsets we observed (e.g., at 555 nm) may become more difficult to discern. Consequently, without clearly defined spectral markers, the current protocol may yield reduced sensitivity and specificity in field settings. As such, to enhance robustness, future research should include higher spectral regions (e.g., 1100–2500 nm), which could increase the chemical information. Further, future studies should build and validate multivariate classification models (e.g., PLS‐DA, random forests) on large, field‐collected training datasets and integrate complementary analytical techniques, such as isotope labeling (e.g., δ^15^N analysis) or metabolomics, to further validate and refine NIR‐based N source differentiation. Although we focused here on *Alnus–Frankia*, the fundamental mechanism—symbiont‐induced shifts in leaf biochemistry and structure—also occurs in other N_2_‐fixing partnerships (Ohyama and Kumazawa 1980). Thus, with appropriate species‐specific calibration, the same NIR approach could be extended to Rhizobia–legume systems, actinorhizal shrubs, or even cyanobacterial associations in ferns. Model retraining using legume spectra, combined with targeted wavelength selection where legume–Rhizobia biochemical signatures are strongest, could allow reliable discrimination of fixation‐derived N in those systems.

## 5 Conclusion

Our findings highlight the potential of NIR spectroscopy as a rapid and nondestructive method for distinguishing between soil‐ and symbiotically fixed N in *A. glutinosa* , which is also more reliable than chlorophyll measurements with a SPAD meter. Despite no apparent characteristic peaks emerging from the spectra of *Frankia* treatments, NIR spectroscopy provides a more precise method to detect slight differences in N that are not detected by conventional chlorophyll measurements using a SPAD meter.

## Conflicts of Interest

The authors declare no conflicts of interest.

## Supporting information


Data S1.

## Data Availability

The data is deposited in Zenodo under (DOI): https://doi.org/10.5281/zenodo.15533926.
