# Supplementary material for: Nondestructive Detection of Frankia in Alnus glutinosa With NIR Spectroscopy
Source: Plant Environ Interact. 2025 Jul 4;6(4):e70066. doi: 10.1002/pei3.70066 (PMC12231197; doi:10.1002/pei3.70066)
Supplement: Supplementary file 1 — Data S1. [file PEI3-6-e70066-s001.docx]

**Supplementary information for**

**Non-destructive detection of *Frankia* in *Alnus glutinosa* with NIR spectroscopy**

Konstantinos Georgopoulos^a,b*^, T Martijn Bezemer^a^, Lars Vesterdal^b^, Kaiyi Li^a^, Léon de Nobel^a^, Sofia IF Gomes^a^

^a^ Above-Belowground Interactions Group, Institute of Biology, Leiden University, Sylviusweg, Leiden, The Netherlands

^b^ Department of Geosciences and Natural Resource Management, University of Copenhagen, Rolighedsvej, Denmark

* Corresponding author: Konstantinos Georgopoulos, Institute of Biology, Leiden University, Sylviusweg 72, 2333 BE, PO Box 9505, 2300 RA Leiden, the Netherlands, [k.georgopoulos@biology.leidenuniv.nl](mailto:k.georgopoulos@biology.leidenuniv.nl)

Keywords: Chlorophyll, Nitrogen, *Frankia*, NIR, spectroscopy

#### **Tables**

**Table S1:** Available soil nutrients of the gamma sterilized soil that is used in all the pots of the experiments expressed in mg/kg soil.

| NH_4_^+^ | NO_3-_ | PO_4_^3-^ |
| --- | --- | --- |
| 37.82±1.61 | 14.41±0.57 | 3.86±0.41 |

**Table S2:** Results from a linear model (LM) testing the effects of *Frankia* inoculation on leaf chlorophyll levels at each week of plant growth starting from week 4. To make the two experiments comparable, the average chlorophyll value of the pure control for each experiment and each week was subtracted from the corresponding chlorophyll measurements of that week. Presented in the table are the degrees of freedom (DF), the F-statistic and the p-value (p). In cases of significant results (p < 0.05), the p-values are bolded.

| Effect | DF | F | p |
| --- | --- | --- | --- |
| Chlorophyll – Week 4  Frankia | 1 | 0.814 | 0.368 |
| Chlorophyll – Week 5  Frankia | 1 | 4.434 | **0.037** |
| Chlorophyll – Week 6  Frankia | 1 | 0.586 | 0.445 |
| Chlorophyll – Week 7  Frankia | 1 | 0.046 | 0.829 |
| Chlorophyll – Week 8  Frankia | 1 | 1.608 | 0.206 |
| Chlorophyll – Week 9  Frankia | 1 | 39.724 | **<0.001** |
| Chlorophyll – Week 10  Frankia | 1 | 73.961 | **<0.001** |
| Chlorophyll – Week 11  Frankia | 1 | 110.000 | **<0.001** |
| Chlorophyll – Week 12  Frankia | 1 | 123.910 | **<0.001** |

**Table S3:** Results from a linear model (LM) testing the effects of fertilizer concentration on leaf chlorophyll levels at each week of plant growth starting from week 4. To make the two experiments comparable, the average chlorophyll value of the pure control for each experiment and each week was subtracted from the corresponding chlorophyll measurements of that week. Presented in the table are the degrees of freedom (DF), the F-statistic and the p-value (p). In cases of significant results (p < 0.05), the p-values are bolded.

| Effect | DF | F | p |
| --- | --- | --- | --- |
| Chlorophyll – Week 4  Concentration | 5 | 2.470 | **0.043** |
| Chlorophyll – Week 5  Concentration | 5 | 2.821 | **0.024** |
| Chlorophyll – Week 6  Concentration | 5 | 7.908 | **<0.001** |
| Chlorophyll – Week 7  Concentration | 5 | 22.472 | **<0.001** |
| Chlorophyll – Week 8  Concentration | 5 | 14.117 | **<0.001** |
| Chlorophyll – Week 9  Concentration | 5 | 15.059 | **<0.001** |
| Chlorophyll – Week 10  Concentration | 5 | 14.682 | **<0.001** |
| Chlorophyll – Week 11  Concentration | 5 | 14.500 | **<0.001** |
| Chlorophyll – Week 12  Concentration | 5 | 18.811 | **<0.001** |

**Table S4:** Results from a one-sample-t-test comparing the aboveground biomass production, leaf chlorophyll and leaf N of trees that received each fertilizer concentration independently against the nodulated control at the time of the harvest. To make the two experiments comparable, the average aboveground biomass, chlorophyll and leaf N of the nodulated control for each experiment was subtracted from the respective measurements of each un-inoculated (pure) fertilizer treatment, setting the nodulated control performance for each measured variable as the 0. P values were adjusted using false discovery rate (FDR). Presented in the table are the t-statistic, the p-value (p) and the FDR adjusted p values. In cases of significant results (p < 0.05), the p-values are bolded. Significant differences from 0 signify lower/higher performance in relation to the nodulated control.

| Fertilizer concentration | t-statistic | p | FDR adjusted p |
| --- | --- | --- | --- |
| Aboveground biomass  0 mM (Control pure)  1.25 mM  2.5 mM  5 mM  7.5 mM  10 mM  20 mM | -15.23  -15.23  -24.46  -12.08  -4.16  1.58  4.31 | **4.19e-12**  **9.84e-08**  **1.52e-09**  **7.25e-07**  **2.44e-03**  0.146  **1.94e-03** | **2.93e-11**  **2.29e-07**  **5.34e-09**  **1.27e-06**  **2.85e-03**  0.146  **2.71e-03** |
| Chlorophyll  0 mM (Control pure)  1.25 mM  2.5 mM  5 mM  7.5 mM  10 mM  20 mM | -24.10  -19.90  -10.25  -9.72  -3.47  -3.80  -1.69 | **1.04e-15**  **9.48e-09**  **2.88e-06**  **4.52e-06**  **7.01e-03**  **4.15e-03**  0.125 | **7.31e-15**  **3.31e-08**  **6.74e-06**  **7.91e-06**  **8.17e-03**  **5.81e-03**  0.125 |
| Leaf N (%)  0 mM (Control pure)  1.25 mM  2.5 mM  5 mM  7.5 mM  10 mM  20 mM | -14.94  -8.59  -34.27  -35.53  -36.26  -17.28  -4.10 | **5.86e-12**  **1.24e-05**  **7.55e-11**  **5.47e-11**  **4.55e-11**  **3.26e-08**  **2.67e-03** | **4.10e-11**  **1.45e-05**  **1.32e-10**  **1.27e-10**  **1.27e-10**  **4.57e-08**  **2.67e-03** |

**Table S5:** The mean ± standard error (SE) of the measured leaf chlorophyll every week after subtracting the mean of the control pure of each experiment from the values. The 0mM uninoculated cell is missing values as that is the control pure.

| Effect | Mean ± SE inoculated | Mean ± SE uninoculated |
| --- | --- | --- |
| Chlorophyll – Week 4  0 mM  1.25 mM  2.5 mM  5 mM  7.5 mM  10 mM  20 mM | 0.56±0.99  -1.16±1.17  0.38±0.77  -1.82±1.08  2.61±0.54  4.50±0.96  4.80±0.81 | X  0.04±0.67  0.95±1.09  1.08±0.55  2.82±0.88  2.81±0.56  3.16±0.97 |
| Chlorophyll – Week 5  0 mM  1.25 mM  2.5 mM  5 mM  7.5 mM  10 mM  20 mM | 0.55±0.81  1.08±0.88  2.07±0.64  1.03±0.71  3.86±0.64  3.53±0.82  4.52±0.91 | X  2.37±0.73  1.78±0.73  2.96±0.79  2.42±0.48  4.24±0.73  4.9±0.78 |
| Chlorophyll – Week 6  0 mM  1.25 mM  2.5 mM  5 mM  7.5 mM  10 mM  20 mM | 0.29±0.94  -0.65±0.90  -0.91±0.69  -1±0.82  5.66±0.90  4.66±0.80  4.31±0.89 | X  -1.41±0.51  0.42±1.18  1.86±1.01  3.32±0.67  4.67±0.62  3.36±0.50 |
| Chlorophyll – Week 7  0 mM  1.25 mM  2.5 mM  5 mM  7.5 mM  10 mM  20 mM | 1.8±0.89  -0.06±0.68  0.88±0.71  -0.14±0.96  6.17±0.85  5.78±1.14  6.87±1.26 | X  -0.06±0.57  0.18±0.75  0.94±1.22  1.97±0.81  5.71±0.96  7.96±0.78 |
| Chlorophyll – Week 8  0 mM  1.25 mM  2.5 mM  5 mM  7.5 mM  10 mM  20 mM | 3.84±1.56  -0.06±0.68  0.88±0.71  -0.14±0.96  5.63±0.98  6.52±0.81  9.54±1.57 | X  -0.06±0.57  0.18±0.75  0.94±1.22  1.97±0.81  5.71±0.97  7.96±0.78 |
| Chlorophyll – Week 9  0 mM  1.25 mM  2.5 mM  5 mM  7.5 mM  10 mM  20 mM | 9.86±1.49  8.74±1.19  6.69±1.13  5.94±1.33  7.85±0.83  8.53±1.13  10.47±2.11 | X  -0.36±1.25  0.37±1.12  0.52±1.34  3.22±0.70  6.42±0.76  10.22±1.12 |
| Chlorophyll – Week 10  0 mM  1.25 mM  2.5 mM  5 mM  7.5 mM  10 mM  20 mM | 13.91±1.43  12.39±1.42  10.20±1.05  8.71±1.39  10.83±1.40  10.32±1.32  12.02±1.58 | X  -0.94±1.04  0.46±1.66  0.74±1.55  5.79±0.85  7.47±0.72  10.91±1.22 |
| Chlorophyll – Week 11  0 mM  1.25 mM  2.5 mM  5 mM  7.5 mM  10 mM  20 mM | 16.96±1.16  17.38±1.01  15.90±0.91  14.54±1.42  15.19±1.58  14.30±1.52  15.11±1.33 | X  0.45±1.12  2.24±1.73  2.98±1.52  9.94±0.96  10.51±0.84  12.99±1.77 |
| Chlorophyll – Week 12  0 mM  1.25 mM  2.5 mM  5 mM  7.5 mM  10 mM  20 mM | 18.83±1.27  20.45±1.65  19.78±1.26  18.05±0.89  17.78±1.31  18.09±1.34  16.17±1.19 | X  -0.3±1.05  1.88±1.82  3.93±1.71  13.16±1.13  12.52±1.19  14.12±1.75 |

#### **Figures**


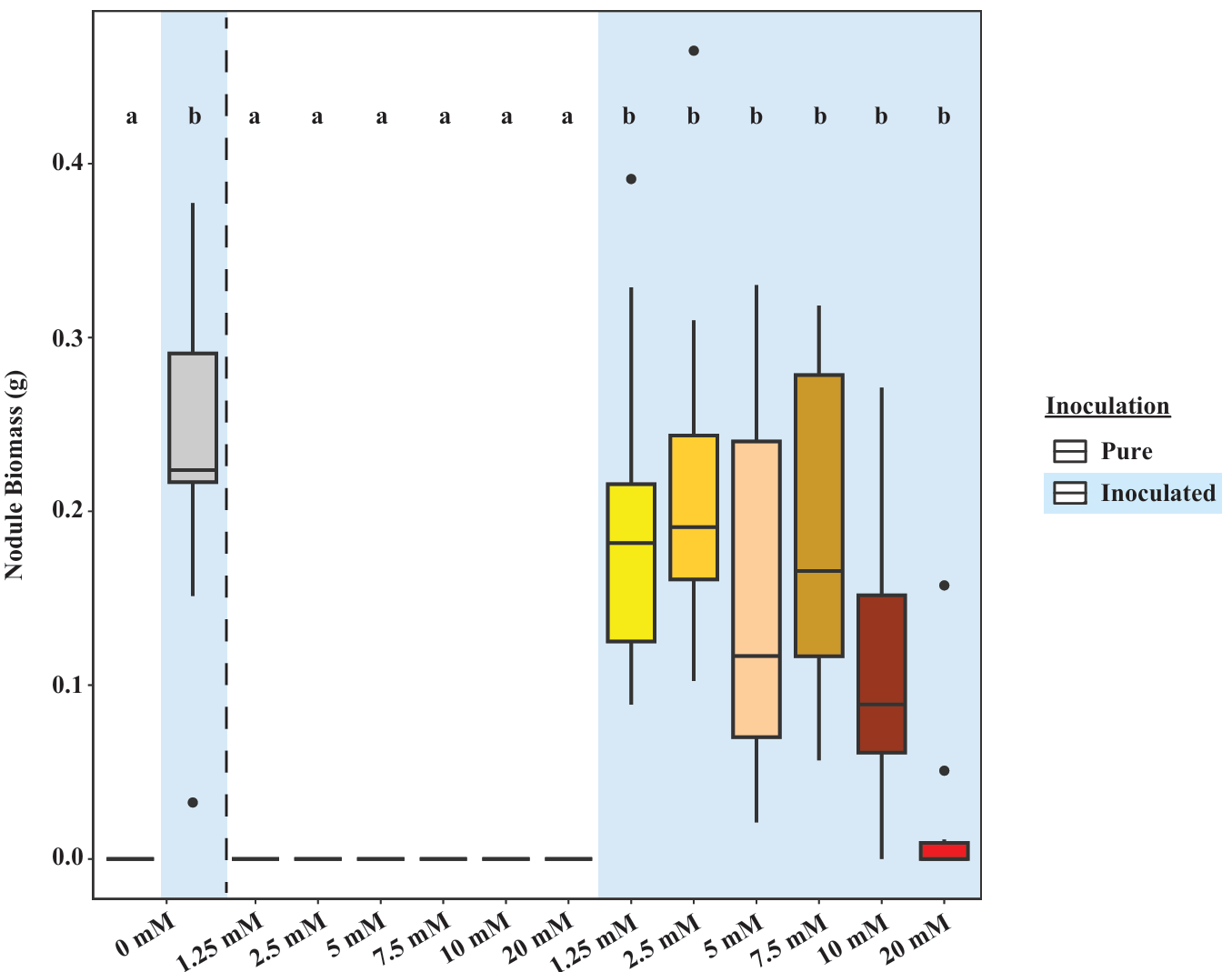


**Figure S1**: The nodule biomass production, of inoculated (blue background) and uninoculated trees that received each fertilizer concentration. Both *Frankia* inoculation and fertilizer concentration had a significant interaction effect on nodule biomass (DF = 6, F = 11.726, p < 0.001). Lettes above the boxplot indicate significant differences calculated with a Tukey post-hoc test. The values were calculated from n= 10 replicates.


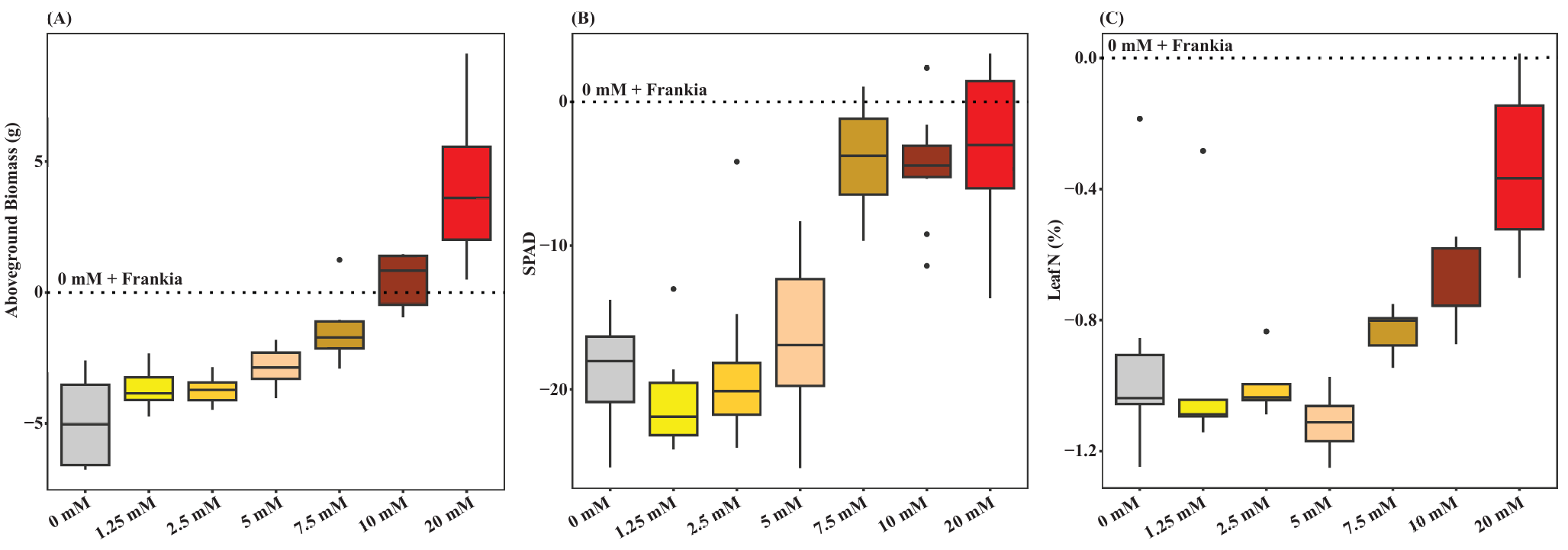


**Figure S2**: (A) The aboveground biomass production, (B) leaf chlorophyll and (C) leaf N of uninoculated trees that received each fertilizer concentration against the nodulated control (dashed line at 0) at the time of the harvest. To make the two experiments comparable, the average aboveground biomass, chlorophyll and leaf N of the nodulated control for each experiment was subtracted from the respective measurements of each uninoculated fertilizer treatment. Statistical outputs from the one-sample-t-test can be found in Table S4.


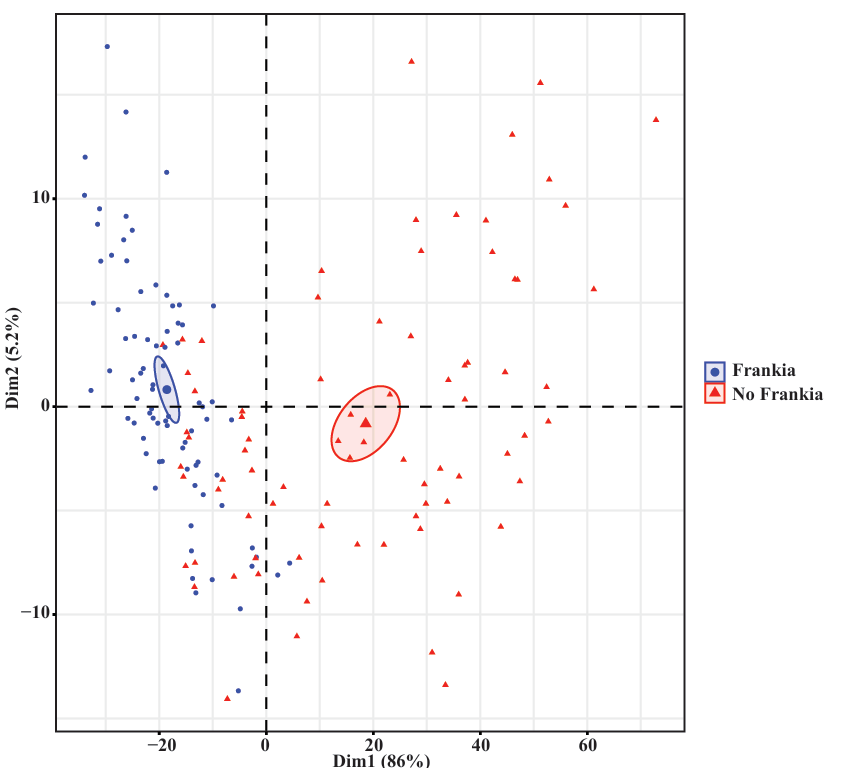


**Figure S3**: The composition of the near infra red (NIR) spectra of the whole dataset (based on Euclidean distances), grouped depending on whether they were inoculated with *Frankia* or not. The ellipses are drawn based on 95% confidence interval.


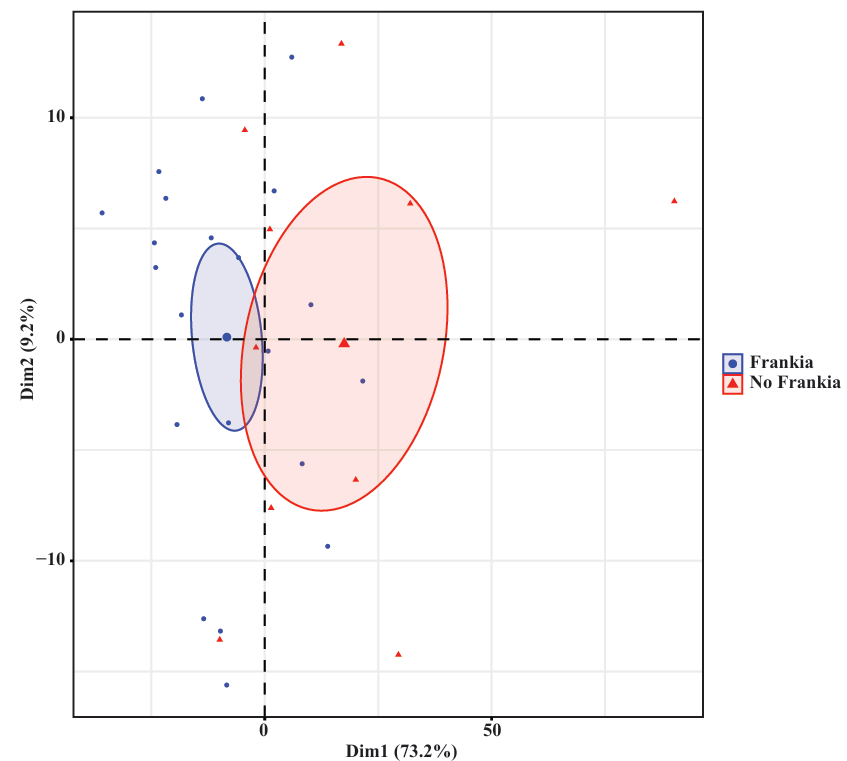


**Figure S4**: The composition of the near infra red (NIR) spectra of only the nodulated control against the similarly performing 20 mM fertilized and un-inoculated treatment (based on Euclidean distances), grouped depending on whether they were inoculated with *Frankia* or not. The ellipses are drawn based on 95% confidence interval.


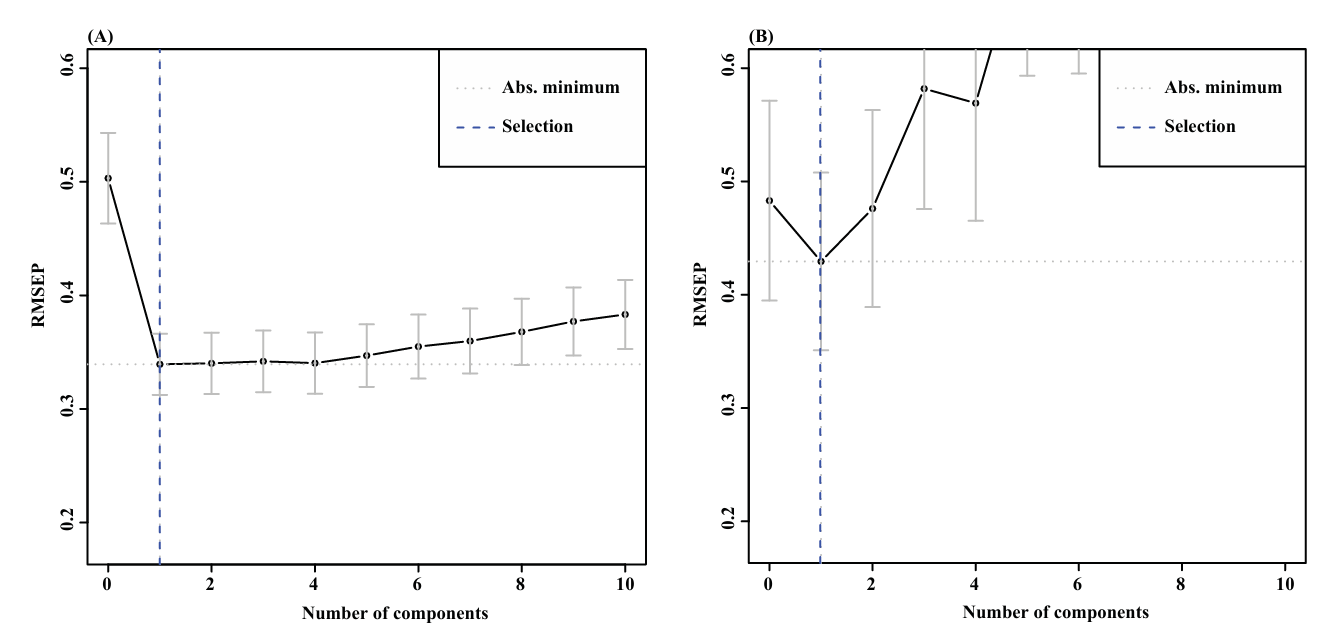


**Figure S5**: The component selection, which was based on the leave-one-out-cross-validated (LOOCV) lowest root mean square error (RMSEP) curves of the PLS model. This was done for (A) all the NIR spectra, and (B) the NIR spectra of only the nodulated control against the similarly performing 20 mM fertilized and un-inoculated treatment. The blue dashed line reveals the selected number of components.


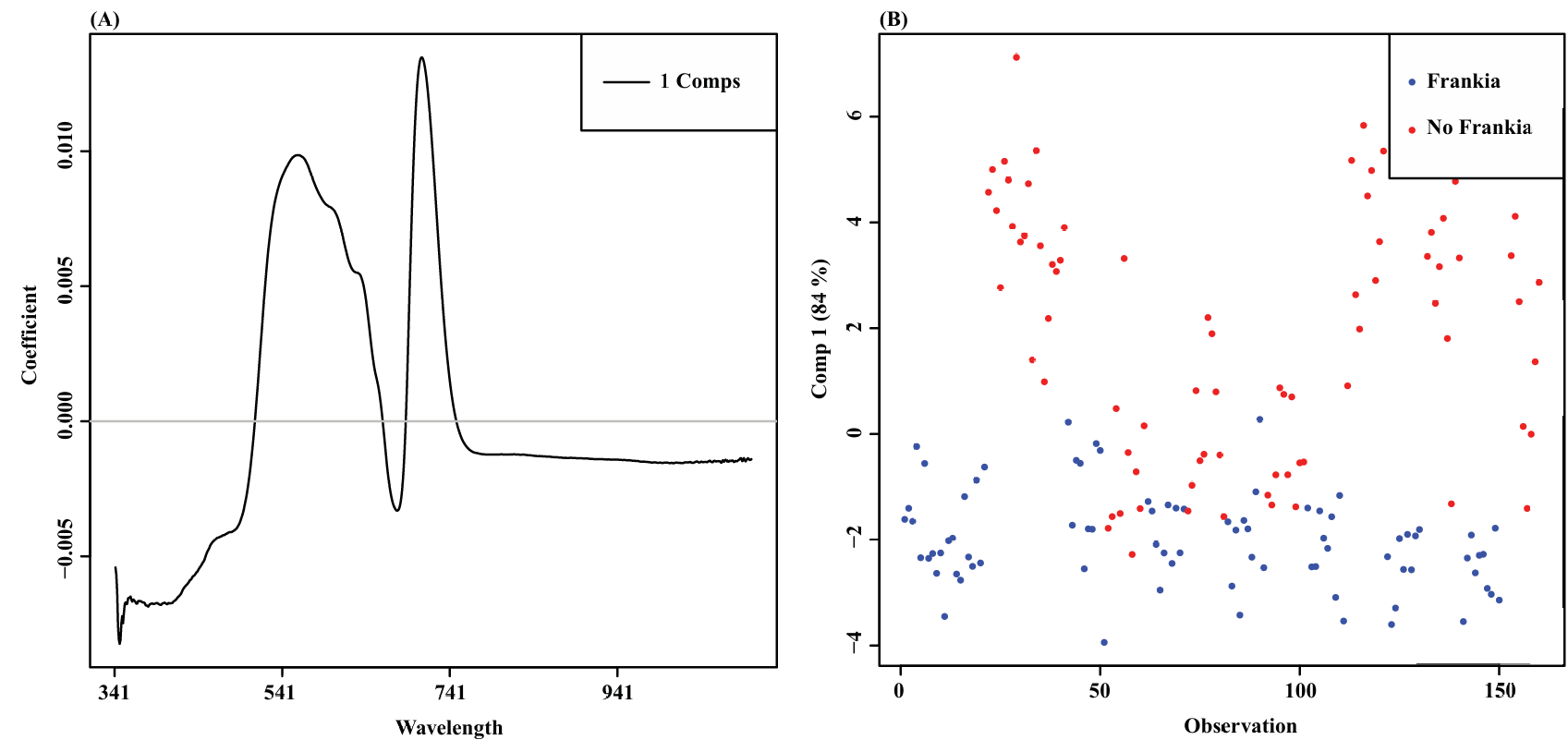


**Figure S6**: The coefficient plot and score plot of the PLS model including the whole NIR spectra.
